# Assessment of critical resource gaps in pediatric injury care in Mozambique’s four largest Hospitals

**DOI:** 10.1371/journal.pone.0286288

**Published:** 2023-06-01

**Authors:** Vanda Amado, Maria Tereza Couto, Manuel Filipe, Jette Möller, Lee Wallis, Lucie Laflamme

**Affiliations:** 1 Department of Global Public Health, Karolinska Institutet, Stockholm, Sweden; 2 Department of the Community Health, Eduardo Mondlane University, Maputo, Mozambique; 3 Department of Surgery, Maputo Central Hospital, Maputo, Mozambique; 4 Mozambique Medical Council Maputo, Maputo, Mozambique; 5 Faculty of Health Sciences, Division of Emergency Medicine, University of Cape Town, Cape Town, South Africa; 6 Institute for Social and Health Sciences, University of South Africa, Pretoria, South Africa; University of Washington, UNITED STATES

## Abstract

**Background:**

Hospitals from resource-scarce countries encounter significant barriers to the provision of injury care, particularly for children. Shortages in material and human resources are seldom documented, not least in African settings. This study analyzed pediatric injury care resources in Mozambique hospital settings.

**Methods:**

We undertook a cross-sectional study, encompassing the country’s four largest hospitals. Data was collected in November 2020 at the pediatric emergency units. Assessment of the resources available was made with standardized WHO emergency equipment and medication checklists, and direct observation of premises and procedures. The potential impact of unavailable equipment and medications in pediatric wards was assessed considering the provisions of injury care.

**Results:**

There were significant amounts of not available equipment and medications in all hospitals (ranging from 20% to 49%) and two central hospitals stood out in that regard. The top categories of not available equipment pertained to diagnosis and monitoring, safety for health care personnel, and airway management. Medications to treat infections and poisonings were those most frequently not available. There were several noteworthy and life-threatening shortcomings in how well the facilities were equipped for treating pediatric patients. The staff regarded lack of equipment and skills as the main obstacles to delivering quality injury care. Further, they prioritized the implementation of trauma courses and the establishment of trauma centers to strengthen pediatric injury care.

**Conclusion:**

The country’s four largest hospitals had substantial quality-care threatening shortages due to lack of equipment and medications for pediatric injury care. All four hospitals face issues that put at risk staff safety and impede the implementation of essential care interventions for injured children. Staff wishes for better training, working environments adequately equipped and well-organized. The room for improvement is considerable, the study results may help to set priorities, to benefit better outcomes in child injuries.

## Introduction

Injury morbidity and mortality are persistent public health problems worldwide(1). The global annual number of deaths reaches 4.4 million people (8% of all deaths), of which 95% occur in Low- and Middle-Income Countries (LMICs) [1,2]. Unintentional Injuries are the leading cause of death among children aged 5 years and older [3].Child injuries are also an important cause of morbidity [4]. Children from resource-poor environments are susceptible to sustaining more severe injuries [5]. Yearly, worldwide more than nine million children are seen in emergency units due to injuries of which the largest proportions in LMICs [6].

In resource-limited settings, a majority of childhood deaths are due to preventable causes, including injuries [7]. Injuries are preventable, e.g., primary prevention measures to prevent injuries from happening [1] or by high-quality care to reduce mortality and morbidity [8]. In health systems in resource-constrained settings, emergency care units are often the weakest section, not only in Africa where significant resource shortages threaten the delivery of essential care [9].

Recently, World Health Organization (WHO) has developed lists of essential resources for emergency care, including required equipment and medication [10]. To the best of our knowledge, the instruments have been employed in three studies involving countries from the Africa region, notably in Sub-Saharan Africa (SSA), and dealing with capacity in emergency surgery at the country level. Studies investigated either one level of care [9,11] or several levels. In the latter case, a study from Cameroon included seven district hospitals, two regional hospitals, two general hospitals, and one missionary hospital [9], whereas another one from Sierra Leone surveyed 10 government hospitals [11] and one from Kenya, 28 facilities across the country [13]. All these studies reported shortages and barriers to care, namely infrastructure for delivering surgical care [11,13] human resources [9], and supplies (like water, electricity, oxygen, and fuel) [12]. In Kenya, where the focus was placed on road-traffic injuries, it was observed that, overall, the facilities surveyed were well-equipped for injuries and geographically relatively not far to reach the facilities in time and distance [13].

Another aspect of concern underlined in the literature on injury care is that healthcare environments and procedures must be adapted to the needs of children [14]. Children are not young adults [15] and routine emergency care interventions may put them at extra risk if they are not properly adapted or tailored to this category of patients (e.g., with short trachea and higher larynx, so intubation requests extra precautions; small bodies that require equipment with appropriate sizes and medication dosages carefully calculated based on weight) [14,15]. Studies mentioned above did not pay attention to those aspects. Moreover, two studies from SSA and LMICs showed the importance to evaluate for children specifically when assessing capacities such as triage, emergency treatment, diagnostic process, identification of co-morbidities, monitoring and supportive care, discharge planning and follow-up, improve training for health workers, to develop preventive measures, to implement an effective trauma system, and to adapt interventions implemented in High-income countries [16,17].

In Mozambique, as in most Sub-Saharan Africa countries, data on pediatric injury care are scarce and there are considerable knowledge gaps regarding acute injury care. In spite of a high and increasing burden of injuries, the country does not have any trauma register, pre-hospital care services, or referral trauma unit or hospital [18]. Recent studies indicate that injuries among children and adolescents are responsible for nearly half of the total burden of hospital injury care [19]. Data from Maputo Central Hospital- Mozambique, 2001, suggest that injuries represent the second leading cause of death in persons aged 15–59 years [20].

This study was embarked upon to gain knowledge on the gaps in pediatric emergency care resources in the hospital settings of Mozambique and on how those gaps, in turn, impede essential injury care. The following research questions were addressed:

1. What equipment and medications are unavailable in the hospitals for pediatric injury care and how do any shortcomings impact essential care?
2. What are the hospital staff perspectives regarding the preparedness of the emergency units for pediatric injury care and what are their priorities for intervention?
3. How well-adapted are pediatric emergency units to child specificity?

## Methods

### Study settings

**Fig 1:**
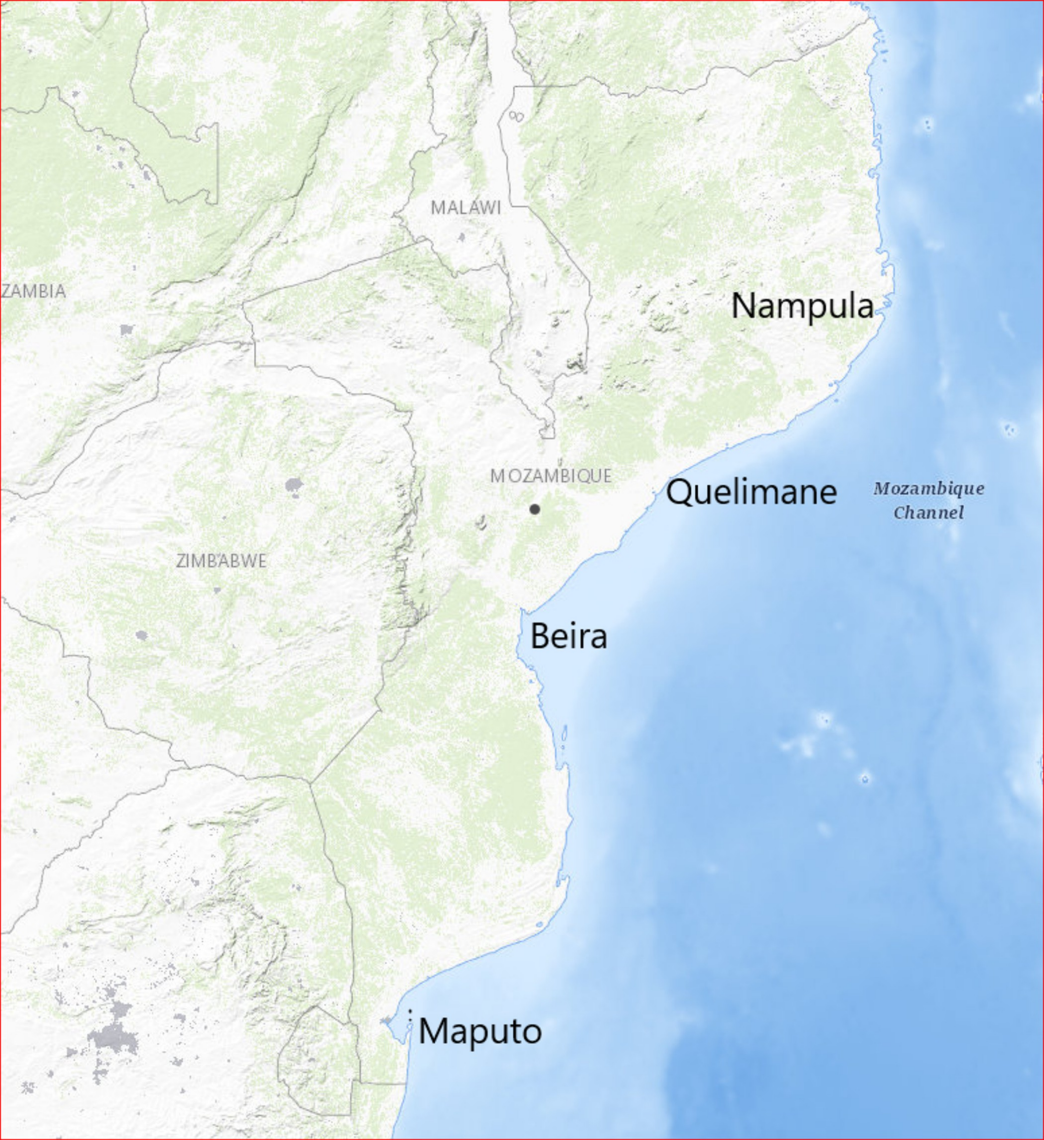
Location of the four central hospitals. Note. Map sourced and adapted from https://apps.nationalmap.gov/viewer/. (Accessed on February 17, 2023).

Mozambique is a low-income country located in south-eastern Africa with an estimated 30 832 244 inhabitants in 2021, of which approximately 45% are children 0–14 years and the median age, is 17.6 years [21]. Mozambique health system is organized in different levels of care: primary health care level (district hospitals and health unit), secondary health care level (provincial and rural hospitals, general hospitals), and finally tertiary health care level (largest hospitals which Mozambique a known as central hospitals). There are four tertiary central hospitals located in three main regions (see Fig 1).

In the south, Maputo Central Hospital (MCH), takes care of children from Maputo city and the provinces of Gaza, Inhambane, and Maputo. It is also the referral hospital of the three central hospitals. The central region has two central hospitals: Beira Central Hospital (BCH), which assists the provinces of Sofala, and Manica. Quelimane Central Hospital (QCH) assists Zambezia and Tete provinces also some districts of Niassa. The north region, Nampula Central Hospital (NCH) assists the provinces of Cabo Delgado, Nampula and Niassa. Whereas the other central hospitals were built over 50 years ago, Quelimane opened in 2019 (See Table 1). In Mozambique, there are private and military hospitals that refer patients to the four central hospitals mentioned above.

**Table 1:** Characteristics of the four central hospitals surveyed in November 2020.

Characteristics	Maputo Central Hospital	Beira Central Hospital	Quelimane Central Hospital	Nampula Central Hospital
Built year	1910	1951	2016	1968
Total number of beds	1 512	1 020	643	607
Pediatric number of beds	307	129	104	192
% Of the total number of beds	21	13	17	32
Total number of injuries per year	35 276	5 563	2 124	7 153
Total hospital staff	4000	1 650	746	1 570
Total emergency staff	225	89	58	92

The four largest hospitals were targeted for this study to provide an overview of the country’s capacity regarding specialized emergency pediatric injury care and highlight potential within-country similarities and differences in that respect.

### Study design

We conducted a cross-sectional study at the country level, targeting Mozambique’s four largest hospitals, including observations, inventories of resources, and administration of a questionnaire among hospital staff.

### Data collection

In November 2020, each hospital was visited by two Mozambican clinicians (pediatric surgeon (VA) and emergency doctor) from the research team to perform data collection. The data was gathered using the following protocol: a first visit guided by local practitioners at pediatric emergency units, standardized and extensive inventory of the equipment and medications in place, self-administrated questionnaire among the hospital staff, and qualitative assessment of child-adaptation of the premises and services. Each visit lasted three days and was performed during work hours from 7:30–15:30 on weekdays. Below is a more detailed description of those different steps.

#### Inventory of equipment and medications

Data on equipment and medications were gathered using the essential equipment and medication lists for emergency care from World Health Organization (WHO), which include two main checklists, for equipment with 248 items and medication with 88 items respectively. Currently, there is no open access to the checklists, but they can be obtained through WHO (emergencycare@who.int). The checklists were not available in Portuguese at the time of data collection; therefore, they were translated from English to Portuguese (the Mozambique official language) and then back translated by a professional translator to ensure fidelity.

The equipment checklist was filled in through direct observations and complementary face-to-face discussions with senior staff in the pediatric emergency unit at each hospital, e.g., head nurse, director of the pediatric emergency unit, and head of the pediatric emergency unit for equipment. As per the WHO instructions, ‘1’ was used for equipment in place and functioning most of the time and ‘0’ for absent equipment (taking note of the reason for the absence e.g., typically not available, ‘broken’ if the item was present but it was not functional and ‘out of stock’ if the item was typically available but currently out of stock at the time of the survey). Similarly, the medication checklist was reviewed with the person in charge of the pediatric emergency pharmacy. The answers related to medication availability were classified according to WHO as: “present and in date”, “present and out of date”, and “absent” if the medication was typically not available. For each hospital, the inventories of equipment and medications were filled in a template uploaded on Microsoft Excel 2013.

#### Staff questionnaire

The staff questionnaire was adapted from the pre-and post-test components of the WHO Basic Emergency Care course [22]. As for the checklists, the questionnaire was translated from English to Portuguese by the researchers and piloted at MCH. In its full version, the questionnaire includes 35 questions, split into three sections: demographics (n = 12), attitudes (n = 14), and knowledge (n = 9). Most questions were multi-choice, and the respondents should indicate their stand, views, or priorities by marking the one or several item(s) that best represented it. The self-administered questionnaire took about 30 minutes to complete. To assess the preparedness of the emergency units for pediatric injury care and what are their priorities for intervention, the following three questions were selected: “what are the most important obstacles to pediatric injury care” (4 obstacles and one open choice), “what priorities should be set to improve that care” (4 priorities and one open choice), and “how prepared is the emergency care unit for dealing with pediatric injury care (2 dichotomous alternatives prepared/unprepared and organized/disorganized; and one open choice).

The directors of each hospital informed the staff when the study would occur, and voluntary participation was emphasized. Of the total 464 emergency unit employees, 313 healthcare workers from the pediatric emergency units (general doctors, surgeons, orthopedics, pediatricians, pediatric and adult intensivists, nurses, and technicians) consented to participate. The overall response rate was 67% (313/464), with some variation between hospitals, Maputo 40%, Beira 76%, Nampula 41%, and Quelimane 60%. Of the 313 respondents, 231 (73.8%) answered the three questions assessing preparedness.

#### Child specificity

In Mozambique as in many other countries, in the clinical setting, child is one aged 0–14 years. To capture the “child-adaptation” of the pediatric emergency unit, the same two clinicians observed for 8 hours (from 7:30–15:30 on a weekday) for 3 days. They observed the infrastructure and equipment in place and how the health workers performed their tasks. The same aspects were systematically observed at each emergency unit. Anything that stood out from the "childcare specificity" was registered, e.g., aspects that complicated the execution of tasks or aspects that could put the health and safety of the child at risk based on the WHO Guidelines for Essential Trauma Care [2]. Further extra observations took place in parallel with the completion of the checklists and while the staff in place was provided time by their employer to fill out the questionnaire. This covered whether the location and organization of the premises facilitated or impeded child-specific treatments and clinical tasks e.g., pediatric injury treatment protocols; whether the tools and equipment were adapted to pediatric metrics e.g., their type and size; how child safety issues can arise, or their wellbeing be threatened given the physical and social environment.

### Data analyses

#### Equipment and medications

Firstly, we quantified how many resources were not available and expressed them in percentages of the total numbers inventoried overall and by the hospital. Therefore, data were then classified the not available resources, categorizing them considering the nature of care that would be impeded or deficient due to the absence, using the WHO Guidelines for Essential Trauma Care [2], which also was a reference for the checklists used. The guideline categorizes the equipment in nine larger categories of care: airway management, breathing (management of respiratory distress), circulation—management of shock, management of extremity injury, management of spinal injury, management of burns and wounds, rehabilitation, pain control, and medicines, diagnosis, and monitoring, safety for health care personnel. Category “others” were introduced for that equipment and medications that did not match any of the essential trauma care categories (e.g., ice source, voltage stabilizer, rescue blankets, table cutter, hospital maps, pre-printed disaster intake forms, backup medical records system if electronic).

#### Staff questionnaire

For each question retained, descriptive statistics of the responses provided by the clinical staff were reported to all hospitals aggregated and by hospital. When applicable differences between hospitals were measured using a Fisher’s exact two-tailed test for differences in proportions due to the small numbers of respondents.

Supplementary data is also provided for two categories of health professionals in all hospitals aggregated: medical doctors (n = 214), nurses/technicians (n = 99).

#### Child specificity

An exhaustive list was compiled of the shortcomings or issues identified in each hospital. For equipment and medication separately, it then thematically analyzed the content of that list to create meaningful categories based on WHO Guidelines for Essential Trauma Care [2].

All statistical analyses were conducted using Stata (Software for Statistics and Data Science, STATA/IC 16.0).

### Ethical approval

This study was conducted following the principles of the Declaration of Helsinki. All methods were carried out in accordance with relevant regulations and guidelines. All participants provide a written informed consent. Participants were informed about the purpose of the study, and researchers are committed to answering their questions. They were guaranteed that the information provided would be kept confidential. In addition, participants were aware that their participation in the study was voluntary and that they could leave the study at any time. When approving the study, the Mozambique Institutional Bioethics Committee of the Faculty of Medicine, and Maputo Central Hospital (CIBS FM &HCM/107/2019) approved the informed consent form.

## Results

### Equipment and medications

Table 2 presents the equipment and medications in board categories and indicates the total number of potential items in each category and the number of missing ones per hospital and, average, all hospital aggregated. For more detailed information about the pieces of equipment or medications included in each category that appeared on the WHO checklists, please see S1 and S2 Tables.

**Table 2:** Number of equipment and medications not available in the hospitals according to functions for essential trauma care, with reference to the WHO checklist (as per November2020).

Resources	WHO Checklist N	Maputo Central Hospital n	Beira Central Hospital n	Quelimane Central Hospital n	Nampula Central Hospital n	Total average n
**Equipment**	248	44 (17.7%)	36 (14.5%)	65 (26.2%)	55 (22.2%)	50 (20.2%)
Diagnose and monitoring	66	9	10	16	11	46
Safety for health care personnel	40	9	6	9	7	31
Airway management	35	3	6	8	10	27
Extremity injury	9	8	1	8	4	21
Fluid resuscitation	15	4	3	4	4	15
Resources for burn and wound	22	3	2	2	5	12
Bleeding control	3	1	1	1	2	5
Breathing/chest injury	2	2	2	1	-	5
Spinal injury	2	-	1	2	1	4
Others (e.g., ice source, voltage stabilizer, rescue blankets, tablet cutter)	139	5	4	14	11	34
**Medications**	88	30 (34.1%)	35 (39.8%)	48 (54.5%)	42 (47.7%)	39 (44.3%)
Infections	16	5	9	13	9	36
Poisoning	11	5	6	6	9	26
Cardiovascular disorders	10	4	3	6	6	19
Anticoagulants and Thrombolytics, including Hemostatic agents	4	4	4	4	4	12
Antipsychotics, barbiturates, sedatives	5	1	4	5	2	12
Corticosteroids	5	3	1	3	4	11
Fluids, electrolyte balance including blood products	9	2	3	3	1	9
Skin diseases: topical applications	5	-	1	3	2	6
Analgesic, antipyretic anti-inflammatory	6	1	-	2	2	5
Vitamins and minerals	5	2	2	1	-	5
Anesthetics	3	-	1	2	1	4
Hormones	3	1	1	-	1	3
Others (e.g., antiemetic, bronchodilator)	6	3	1	1	2	7

* Categories presented in decreasing order of non-availability.

As indicated in Table 2, there was equipment and medications not available in all four largest hospitals. In total, 20.2% of equipment and 44.3% of medications were not available at least in one hospital. Quelimane and Nampula hospitals had a higher average of missing equipment, with 26.2% and 22.2% respectively, compared with Maputo (17.7%) and Beira (14.5%) hospitals. Those two hospitals also presented a higher percentage of not available medications (54.5% and 47.7% respectively), compared to Maputo (34.1%) and Beira (39.8%).

### Nature of the not available equipment and medications

By, far, the largest category of equipment not available was related to diagnosis and monitoring (n = 46 in total, e.g., eco-doppler, otoscope, ophthalmoscope, flashlight and pregnancy urine test), particularly in Quelimane, equipment to ensure the safety of health care workers (e.g., environmental disinfectant, body bag, fans, shoe covers) and those for airway management (e.g., oxygen mask of neonatal sizes, pediatric bag–valve–mask of neonatal sizes, CPAP, BiPAP neonatal mask and endotracheal tubs). In addition, two hospitals, Maputo and Quelimane, lacked several types of equipment for the treatment of extremity injuries (e.g., splint material such as, stocking, gauze padding, premade splint, tension or traction splint, and plaster cast remover).

Three categories of medications stood out for not being available in all hospitals: 1. medications to treat infections (e.g., antibiotics for lung and skin infection, IV antifungal and rabies vaccine) (n = 36 in total), most strikingly so in Quelimane (n = 13); poisoning medications (n = 26; e.g., diphenhydramine, neostigmine, an antidote for lead exposure); tablets to treat cardiovascular disorders (n = 19); e.g., IV diuretics, advanced vasopressor support).

### Child specificity on local facilities and premises in the pediatric emergency unit

As indicated in Table 3, there were several issues about the adequacy of equipment and medications concerning pediatric emergency care. Regarding the equipment, pertained to missing equipment, in particular the absence of an infrastructure for injury care in all pediatric emergency units, including the lack of room for minor interventions (e.g., sutures, plaster placement, and fracture reduction), operation for laparotomy, radiological examination (e.g., X-ray, Computed Axial Tomography (TAC) and ultrasound). Also, albeit available in the pediatric units, some essential equipment was not of appropriate size for pediatric patients (e.g., urinary catheter, chest tube, cervical collar, and nasogastric tube). Except for MCH, we found that pediatric emergency departments lacked essential procedures equipment that otherwise was available for adult emergencies (often located very far from the pediatric ones), like surgical cricothyroidotomy set and basic immobilization equipment. Finally, some equipment was available but was not used for reasons like the absence of reagents for laboratory equipment to do arterial blood gas measurements or the lack of power current stabilizer, battery for otoscope, and laryngoscope.

**Table 3:** Type of shortcomings observed in November 2020 as regards the child specificity of pediatric emergency.

Shortcoming	Example
***Equipment***	
Available but not in pediatric ward	Surgical procedure room, room for radiological examinations such as x-ray, TAC, Ultrasound.
Available but not of size adapted to pediatric patient	Urinary catheter, chest tube, cervical collar, nasogastric tubes
Not available equipment for some pediatric surgery procedures	Surgical cricothyroidotomy set, anal speculum, Basic immobilization (sling, splint)
Available but not usable	Absent of reagents for laboratory equipment to do arterial blood gas measurements or lack of electric power stabilizer, battery for otoscope, laryngoscope
***Medications***	
Not available instructions to administer correct dosage of emergency medication to pediatric patients	Appropriate references or charts to calculate pediatric doses not available/provided
Inappropriate size of packaging, involving safety risks	Packaging of large volumes of intravenous saline (one litter of saline solution)

As for medications, all hospitals lacked support tools for giving medication to pediatric patients, e.g., appropriate references or charts to calculate pediatric doses. Also, the medication available was in packages of inappropriate sizes, involving safety risks e.g., packing large volumes of intravenous solution as one liter of saline solution.

Finally, most of the time, severely injured pediatric patients were seen by general doctors, general surgeons, emergency adult doctors and sometimes with the technician.

### Staff perception of needs and priorities

**Fig 2:**
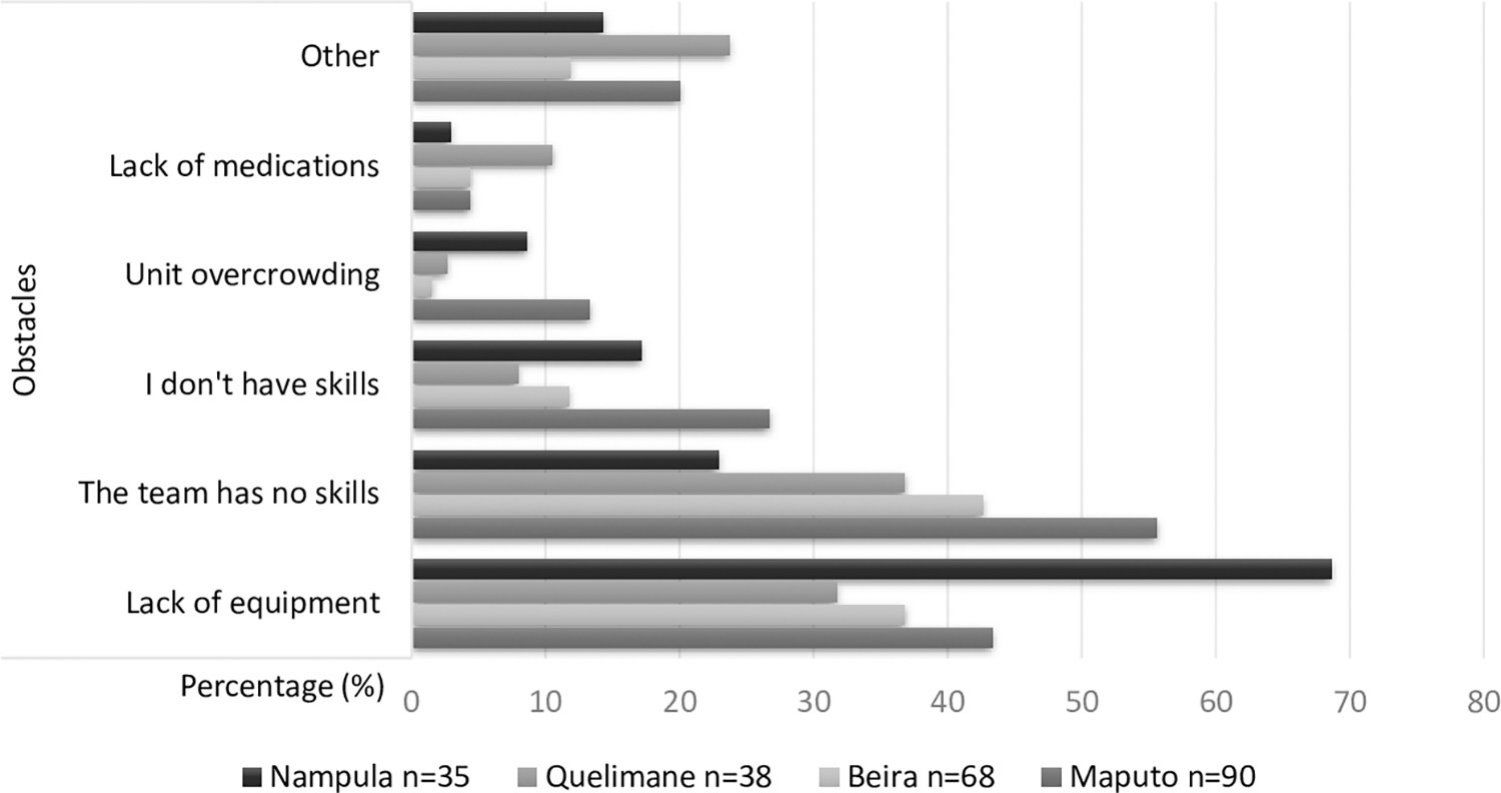
Principal obstacles in the emergency unit for pediatric injury care according to the clinical staff of each hospital, answers presented in percentages (%).

**Fig 3:**
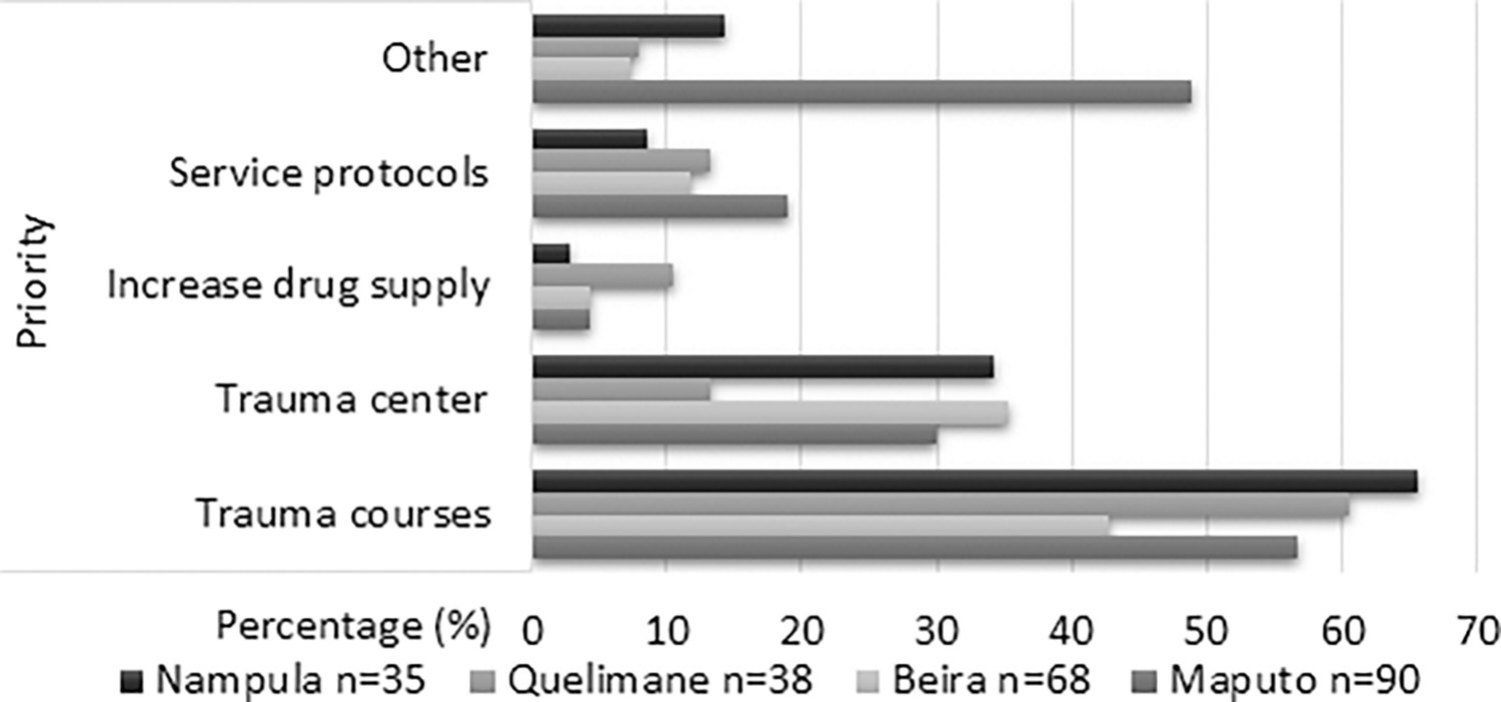
Principal priorities in the emergency unit to improve pediatric injury care according to the clinical staff of each hospital, answers presented in percentages (%).

**Fig 4:**
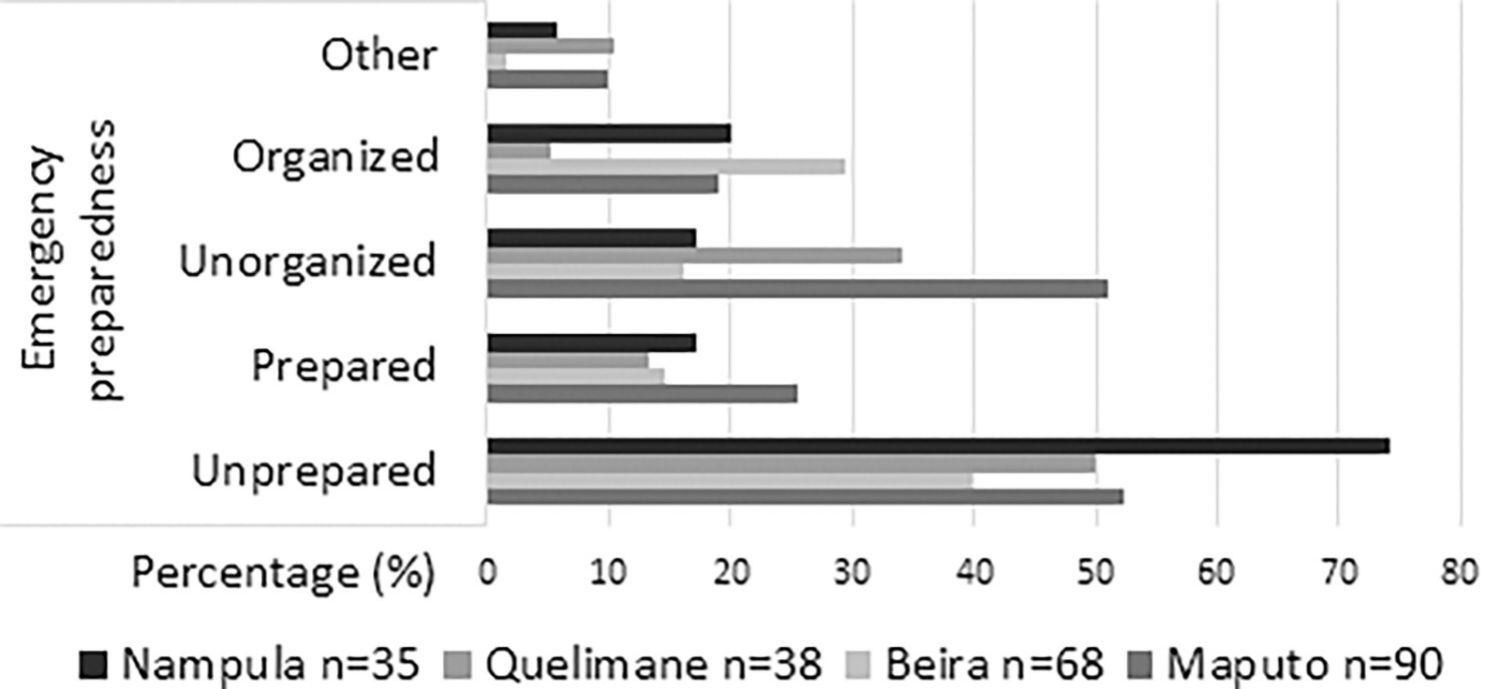
Preparedness for pediatric injury care in the emergency unit according to the clinical staff of each hospital, answers presented in percentages (%).

Table 4 presents how the staff at each hospital, all occupations aggregated, answered the three questions concerning, in turn, principal obstacles and priorities to improve injury care, and the preparedness of the emergency unit as regards pediatric injury care (see also Figs 2 to 4).

**Table 4:** Opinions of the emergency unit clinical staff concerning obstacles and priorities to improve injury care as well as the preparedness of the unit, all hospitals aggregated and by hospital.

	All hospitals n = 231		Maputo Central Hospital n = 90		Beira Central Hospital n = 68		Nampula Central Hospital n = 35		Quelimane Central Hospital n = 38	
**Main obstacles to adequate pediatric injury care**										
	**n**	**%**	**n**	**%**	**n**	**%**	**n**	**%**	**n**	**%**
Lack of equipment	100	43.3	39	43.3	25	36.8	24	68.6	12	31.6
The team has no skills	101	43.7	50	55.6	29	42.6	8	22.9	14	36.8
I don’t have skills	41	17.7	24	26.7	8	11.7	6	17.1	3	7.9
Unit overcrowding	17	7.4	12	13.3	1	1.5	3	8.6	1	2.6
Lack of medications	12	5.2	4	4.4	3	4.4	1	2.9	4	10.5
Other	40	17.3	18	20.0	8	11.8	5	14.3	9	23.7
**Priorities to improve pediatric injury care**										
	**n**	**%**	**n**	**%**	**n**	**%**	**n**	**%**	**n**	**%**
Trauma courses	126	54.5	51	56.7	29	42.6	23	65.7	23	60.5
Trauma center	68	29.4	27	30.0	24	35.3	12	34.3	5	13.2
Increase drug supply	12	5.2	4	4.4	3	4,4	1	2,9	4	10.5
Service protocols	33	14.3	17	18.9	8	11.8	3	8.6	5	13.2
Other	57	24.7	44	48.9	5	7.4	5	14.3	3	7.9
**Emergency units’ preparedness for pediatric injury care**										
	**n**	**%**	**n**	**%**	**n**	**%**	**n**	**%**	**n**	**%**
Unprepared	119	51.5	47	52.2	27	39.7	26	74.3	19	50.0
Prepared	44	19.0	23	25.6	10	14.7	6	17.1	5	13.2
Unorganized	76	32.9	46	51.1	11	16.2	6	17.1	13	34.2
Organized	46	19.9	17	18.9	20	29.4	7	20.0	2	5.3
Other	16	6.9	9	10.0	1	1.5	2	5.7	4	10.5

For the obstacles (see also Fig 2), except for the “lack of equipment”, the answers are quite similar across hospitals. The staff from Nampula stands out with as many as 68.6% of the staff considering that the equipment is an obstacle for the administration of pediatric emergency care, which is much higher than in the other hospitals.

For the priorities (see also Fig 3), a significant difference arises only in the case of trauma center where almost one-third (35.3%) of the staff from Beira regarded it as a priority for pediatric trauma care.

By contrast, there are several differences when it comes to how prepared and organized the local emergency injury care is perceived (Fig 4). The clinical staff from Nampula expresses its agreement with unpreparedness to a far greater extent than the staff from other hospitals (74.3%), followed by Quelimane (50.0%). The clinical staff from Maputo and Quelimane expressed the view that the unit is unorganized (51.1% and 34.2%) to a greater extent than the staff at Beira (16.2%) and Nampula (17.1%) and, by contrast the clinical staff from at Beira stands out as more incline to see the unit as organized (29.4%) and that of Quelimane, not to (5.3%).

Stratification of the answers by the occupational group’s medical doctors and nurses/technicians (see S3 Table; headings Doctor and Nurse respectively) show a number of significant differences within hospitals, but we must also take into consideration the small numbers of responding nurses and technicians in Beira and Quelimane. Looking at Maputo hospitals where the numbers are higher, one can see that lack of equipment is seen as an obstacle in high proportions by both groups but significantly more so among doctors (67.6% and 47.6% respectively). The proportions of doctors considering the lack of skills in the team as an obstacle is also significantly higher (46.7% compared to 16.7%), just as in Beira (50.0% compared to 15.8%). By contrast, significantly more nurses/technicians than doctors would prioritize trauma courses to improve pediatric trauma care (47.6% and 29.7% respectively). Still at Maputo, a great proportion of doctors (64.8%) than nurses/technicians (33.3%) consider the emergency unit unprepared for pediatric injury care.

## Discussion

### Main findings

The amount of pediatric injury care equipment and medications not available in the country’s four largest hospitals was noteworthy. While regional differences arise, to the detriment of the North region, all hospitals faced issues that either put at risk staff safety or impeded the implementation of essential care interventions of injured children. In the latter case, the common unavailable equipment related to diagnostic and monitoring or airway management and medications to treat infections and poisoning. Further, in many instances, injured children received their first hospital care in the adult emergency unit rather than in the pediatric one, where the staff is better trained and better prepared to treat children and where infrastructure, equipment, and medications, when available, are more likely to be adapted to this patient group. Finally, the emergency care personnel from those hospitals expressed concerns primarily about the staff skills in pediatric injury care and the lack of equipment and organization of the emergency unit.

Our results on the number and nature of missing equipment and medications and concerns regarding staff training find an echo in the few other large studies using the WHO instrument either country-specific in the Republic of Cameroon [9] Sierra Leone [23]; Kingdom of Eswatini [24] or multi-country [25]. In the Republic of Cameroon, where hospitals from different regions and at several levels were surveyed, substantial gaps in equipment allowing for interventions in the areas of diagnosis and monitoring, equipment for the management of special injuries, and equipment for airway and breathing were noted [9]. Also, in the Kingdom of Eswatini [24], the emergency unit of the three governmental referral hospitals surveyed lacked medications (e.g., thrombolytics) and equipment (for the diagnosis and monitoring (e.g., electrocardiograms and ultrasound) and were impeded in their ability to perform some investigations due to lack of reagent stock. Further, in Sierra Leone, 10 of the 17 government civilian hospitals surveyed for their surgical capacity had shortcomings in their availability of specialized human resources and basic resources like electricity, running water, oxygen, and fuel. The above contrasts with the observations made in 28 hospitals in Kenya when focusing on road traffic injuries and reporting both well-equipped and easily accessible facilities [13].

Even in the multi-country study (LeBrun et al [26] where 7 LMICS were surveyed (of which 3 were African countries), from the total of 78 government district hospitals the authors showed that there were insufficient and inequitable distributions of trained surgical providers, inadequate infrastructure, and shortages of essential equipment so they were not equipped to address surgically treated conditions.

To the best of our knowledge, staff perspectives on pediatric emergency care have not been much researched. However, our results are like the study of the governmental referral hospitals from the Kingdom of Eswatini [24] that revealed problems in the emergency care organization (such as lack of protocols for flow through emergency care areas) and gaps in training related to critical trauma care, airway interventions, and neonatal care.

It is also of note that the country suffers an important economic divide between rural and urban areas, to the detriment of the latter. This poverty may explain, at least in part, why Quelimane, albeit new, remains under resourced.

The shortcomings that we observed regarding the equipment and medication for the treatment of injured children have not been reported in the studies mentioned above [24]. Yet, a range of considerations have been put forward for pediatric emergency care in resource-poor settings that raise issues echoing the ones found here; namely, mal-adapted equipment, medication lacking pediatric formulation, or available in adult-dose tables, not available guidelines, and lack of training [17]. In addition, in the African context, a previous study emphasized that injured children from most countries of the continent are treated by medical professionals (e.g., generalists or general surgeons) with no trauma or pediatric surgical training—or even by non-physicians [21].

Across LMICs, health ministry’s face a range of quantitative and qualitative challenges in health and hospital care delivery. Governments need to prioritize the allocation of resources and investments. The framing of global health funding means that system priorities are often in vertical programs or driven by funders and, as a result, priority is given to, for example, HIV care, leaving services for non-communicable diseases largely unattended. In this study, this translates into a range of equipment and medications of importance for trauma care missing even in specialized hospitals.

Highlighting trauma care as a priority for LMIC health ministries is an on-going challenge, despite the obvious–and growing–injury burden. Advocacy remains key but must be supported by robust data, a large part of the motivation behind this study. Training pre-service health workers on this often-overlooked disease burden is also key, along with the introduction of post-graduate and specialist training opportunities.

## Strengths and limitations

One strength of the present study is that it covers the entire set of the main and referral hospitals in the country. These are the only hospitals where specialized in emergency trauma care in general, and pediatric, was provided at the time of the investigation. In that sense, the observations made reflect the situation–in its best possible form–at the country level. Whether hospitals of that level of care are not available to the population in general (like private hospitals or military ones of care) would perform better remains to be determined. But as the type of care, we investigate is quite specialized and child-specific, there are no reasons to believe that those other hospitals would focus on pediatric patients an additional strength lies in the fact that the study uses two complementary and standardized data collection instruments [2,10]. This allows for an appropriate description and understanding of the impact of the missing equipment and medications on trauma care, alongside a more in-depth child perspective approach.

One important aspect of the data collection process is that it actively involved the clinicians in the gathering of timely data within the hospital setting. This allowed for the incorporation of local sensitivities, willingness to participate through local trust, and validation of the process with the health professionals.

However, as the study is cross-sectional, it is not easy to determine how representative our time window is of any other one during the year or over time. Eventually, some missing equipment and medications could have been available before or after the site visits. But, given the level of unavailability observed and the comments from the staff, it is doubtful that the picture would differ substantially at another point in time. It is of note that, despite costs inherent to the conduct of this study in a country as large as Mozambique, all hospitals targeted were visited and surveyed. Whether follow-ups will be feasible remains to be determined.

The study was conducted during the first year of the Covid19-pandemic, at the end of 2020. To some extent, social distancing rules in place delayed and slowed down the data collection process and might have impeded to some extent our access to the hospital staff. Eventually, it contributed to a lower response rate among the staff.

The structure of the hospitals and services care delivery in the country’s largest hospitals were another limitation or source of worry. Pediatric care as well as specific children-related procedures have been mixed with those of adults, making it difficult to get specific children-related answers from health professionals as the services are spread and non-specific. There are many geographically spread services across many departments within the hospitals, which in some cases complexified the data collection, however, all spread services were visited, and the staff was interviewed.

## Implications of the study

The study indicates that the central hospitals of Mozambique are not adequately equipped and lack medications to deal with pediatric trauma patients. Given that the health care system of the country must deal with a range of health conditions, several with their own specific needs [27], how best to redress the situation remains to be studied. To tackle this, not only the circumstances of the health care providers will deserve consideration but also those of the children, who are not small adults, have special needs, and represent a large proportion of the population of the country. Therefore, it is highly desirable that pediatric wards are provided with the resources needed for timely and adapted trauma care and there is a case for greater attention to be paid to critical childcare, including needs assessment, prognostication, and cost-effectiveness [28]. As indicated by the results, guidelines and protocols for emergency trauma care are needed, which are known factors susceptible to benefit health outcomes.

Whether trauma care services should coexist with other activities within the hospitals [9,12] and whether triage systems should be revisited are additional questions that need closer consideration. The point has been made those horizontal interventions, i.e., the ones that benefit several diseases at the same time may provide better services/care than vertical ones, such as strengthening surgery. That said, the provision of emergency trauma care in the country needs to be revisited, for instance, by making better use of some capacities locally available [29]. This could be extended to subnational level hospitals (different from the central ones). Finally, how best to train staff and what skills need to be prioritized remains to be determined [30,31].

## Conclusions

The amount of pediatric injury care equipment and medications not available in the country’s four largest hospitals were considerable and there were worrisome life-threatening shortcomings in how the special needs of children were taken into consideration in all hospitals. Albeit to a different degree, all four central hospitals faced issues that put at risk staff safety and impeded the implementation of essential care interventions for injured children. Acknowledging these are important, local staff wishes for better training, adequately equipped and well-organized working environments. The room for improvement is considerable and the results of the study may help set priorities, to the benefit of better outcomes in child injuries.

## Supporting information

S1 Table Equipment categories according to functions for essential trauma care, with reference to the WHO checklist. (DOCX)

S2 Table Medications categories according to functions for essential trauma care, with reference to the WHO checklist. Note. From the medication checklist, was removed item nine about obstetric emergency units.
(DOCX)

S3 Table Opinions of the emergency unit clinical staff concerning obstacles and priorities to improve injury care as well as the preparedness of the unit, stratified by hospital and occupational group. (DOCX)
