# Supplementary material for: Assessment of critical resource gaps in pediatric injury care in Mozambique’s four largest Hospitals
Source: PLoS One. 2023 Jun 1;18(6):e0286288. doi: 10.1371/journal.pone.0286288 (PMC10234533; doi:10.1371/journal.pone.0286288)
Supplement: S1 Table — (DOCX) [file pone.0286288.s001.docx]

**Supporting information**

**S1 Table:** Equipment categories according to functions for essential trauma care, with reference to the WHO checklist

| **Categories for essencial trauma care** | **WHO Checklist equipment** | | |
| --- | --- | --- | --- |
| **Diagnose and monotoring (66)** | Ultrasound Endo cavitary probe (3.5 - 11.5mHz) | Quality controls for glucometer | Fluid collection bag |
|  | Ultrasound gel | Finger-stick lancets | Tubing and connectors for needle procedure drainage |
|  | Ultrasound machine | Glucometer | Doppler |
|  | Ultrasound probe covers | Urine dipstick | Spinal needles for lumbar puncture |
|  | Ultrasound probes [e.g., curvilinear probe (2 - 5mHz), linear probe (3.5 - 9.5mHz), phased array probe] | Urine pregnancy test | Ophthalmoscope |
|  | Electronic cardiac monitor | Malaria rapid diagnostic test | Drainage bag for catheters, drains |
|  | Electronic cardiac monitoring leads with suction attachments | Point of Care Hemoglobin/Hematocrit testing | Vaginal speculum |
|  | Pediatric length-based tape (e.g. Broselow tape) | POC laboratory equipment | Drainage bag for catheters, drains |
|  | Child weighing scale (250g graduation) | HIV rapid testing kits | Esophageal detector device (e.g., CO2 colorimeter) |
|  | Pulse oximetry | Specimen containers | Diaper (adult and child) |
|  | Measuring tape or marked object to measure children (e.g., length, head circumference, MUAC) | Sample transport containers (cold box) | ECG machine |
|  | Clock, timer or watch with second hand | Laboratory tubes, safe transfer devices and labels | ECG paper, leads and suction attachments/stickers |
|  | Stethoscope | Specimen bags for safe transport | X-ray viewing box or digital system |
|  | Digital thermometer (32 - 43 Celsius) | Sterile specimen containers | Portable x-ray machine/c-arm |
|  | Penlight | Culture bottles (aerobic and anaerobic) | Insulin needles and syringes (safety, single use) |
|  | Lubricating jelly | Anal speculum | Urinary catheter (5 - 22F) |
|  | Magnifying loupes | Long catheter-sheathed needles for paracentesis, ultrasound-guided venous access, etc. (e.g., 18G, 7.5 - 10 cm) | Urinary straight catheter |
|  | Pediatric sizes for blood pressure cuffs | Magnifying loupes | Urinals |
|  | Otoscope tips | Exam lighting (fixed) | Bed pans |
|  | Otoscope | Headlamps | Sanitary pads |
|  | Tongue depressors | X-ray viewing box or digital system |  |
|  | Ear Curette | Portable x-ray machine/c-arm |  |
|  | Glucometer test strips | Fluid collection vessel |  |
|  | | | |
| **Safety for health care personnel (40)** | Death kit (any need documentation, tags, or labels for preparing dead patient for transport to morgue) | Cleaning protective equipment (e.g., thick gloves, boots) | Sharps disposal (single use cardboard box or locked plastic box; puncture proof; lid closes when 3/4 full) |
|  | Isolation setup (e.g., tent, designated room) | Cleaning equipment (e.g., sponges, buckets, mops, brooms) | Red and yellow plastic bags |
|  | Goggles | Hair covers | Rubbish bags |
|  | Aqua tabs (or equivalent) | Environmental disinfectant | Safe biological waste disposal containers |
|  | Mosquito nets | Mask with face shield | Water treatment mechanism (chlorine, tester) |
|  | Fans | Surgical mask | Safe final disposal of biological waste |
|  | Chairs | N-95 respirators | Safe final disposal of sharps |
|  | Tables or desks | Impermeable aprons | Body bag |
|  | Patient tags (e.g., bracelets) | Non-sterile gowns (multiple sizes) | Portable air filtration system |
|  | Rescue blankets | Sterile gowns (multiple sizes) | Decontamination pack |
|  | Fire extinguishing mechanism (e.g., blankets, extinguishers) | Autoclave (electric or non-electric) and supplies or source of sterilized procedure sets | Sheltered surge space (at least two separate spaces for living and dead; not existing clinical area spaces) |
|  | Heating or cooling units | Shoe covers | Signage for area/function designation |
|  | Soap | Hand disinfectant | Patient slide for stretcher transfers |
|  | Sterile gloves (size 6 - 8) | Environmental disinfectant |  |
|  | | | |
| **Airway management (34)** | ENT set | Nebulizer | Oral airways (#00 - 6) |
|  | Pediatric size Magill forceps | Laryngoscope set (range of blades and sizes) | Nasal airways (12 - 36F) |
|  | Dual-tube laryngeal mask airway (LMA) (#2 - 7) | Nasal prongs (adult, pediatric and neonatal sizes) | Bag–valve–mask (adult, pediatric and neonatal sizes) |
|  | Mechanical Ventilator | Nebulization masks (pediatric and adult sizes) | PEEP valve for bag-valve-masks |
|  | Circuit/tubing for mechanical ventilation | Nebulizer tubing and connectors | Capnometry |
|  | CPAP, BiPAP neonatal mask | Metered-dose inhaler spacer | Suction catheters |
|  | CPAP, BiPAP child mask | Peak-flow meter | Suction device: manual (bulb) or foot pump |
|  | Continuous or bi-level positive airway pressure machine (CPAP, BiPAP) | Oxygen supply (cylinder, concentrator, or other source; with flow meter) | Suction device: powered (electric or pneumatic) |
|  | Pediatric sizes for laryngoscope | Tracheostomy tubes (4 - 10mL inner diameter) | Suction tubing (#10 - 16) and connectors |
|  | Endotracheal tubes (#2.5 - 8.5) | Oxygen mask (adult, pediatric and neonatal sizes) | Yankauer or other stiff suction tip |
|  | Bougie | Non-rebreather |  |
|  | Surgical cricothyroidotomy set | Oxygen tubing and connectors |  |
|  | | | |
| **Extremity injury (8)** | Plaster cast remover | Splint material (i.e. - stocking, gauze padding, premade splint) | Crutches |
|  | Plaster of Paris or equivalent | Tension or traction splint (e.g., hare splint) | Physical Restraints |
|  | Sheet or binder for pelvic fractures | Elastic bandages |  |
|  | | | |
| \| **Fluid resuscitation (15)** \| \| --- \| | IV infusion flow regulator (manual with dial) | Intraosseous needle or equivalent (15, 25 and 45mm) | Blood administration set |
|  | Intravenous infusion set (lines, connectors and cannulas size 14 - 24) | Fluid warmer | Syringe (2, 5, 10, 20, 50cc) |
|  | IV poles or hooks | Pressure bag for IV infusion | Syringe (60cc catheter tip) |
|  | Tourniquets for IV start | Rapid infusion catheter set | Needles (range of gauges and lengths; safety, single use) |
|  | Intraosseous needle driver (electric or manual) | Seldinger technique kit for central line placement & fluid drainage (e.g. - pericardial/pleural effusion, ascites) | Autotransfusion set |
|  | | | |
| **Resources for burn and wound (23)** | Low-acuity grab bag (e.g. dressing supplies) | Sutures (absorbable and non-absorbable; sizes 2-0 - 6-0) | Stretchers and gurneys (wheeled stretchers) |
|  | Kidney basins | Minor surgical set | Cotton wool |
|  | Trauma shears | Sterile dressings | Wheeled procedure tray table |
|  | Sterile procedure drapes | Vaseline or paraffin gauze | Povidone iodine 10% solution |
|  | Non-sterile procedure drapes | Dental set | Chlorhexidine 5% solution |
|  | Non-sterile dressings | Sterile dressing set | Ethanol 70% solution |
|  | Scalpel | Procedure (kick) bucket | Razor |
|  | Adhesive tape | Non-sterile dressings |  |
|  | | | |
| **Bleeding control (3)** | Cautery pens | Nasal tampons or equivalent | Arterial tourniquet |
|  | | | |
| **Spinal injury (2)** | Paediatric sizes for cervical collars | Burr hole set |  |
|  | | | |
| **Other (34)** | Outdoor lighting | Defibrillator with pacing and synchronized cardioversion capabilities | Extension cables |
|  | Maps of hospital | Defibrillator (with manual controls) | Voltage stabilizer |
|  | Maps of local area | Defibrillator pads | Folding camp cots |
|  | Refrigerator with thermometer | Oral medication administration supplies (e.g., cups, dropper) | Back up high acuity grab bag |
|  | Ward screens | Medication dispensing containers and labels | Low acuity grab bag (e.g. dressing supplies) |
|  | Lockable box for controlled medications | Stocked high acuity grab bag | Backup medical records system if electronic |
|  | Medication and medication dispensing storage and organization system | Triage tags | Backup patient tracking system (e.g. white board) |
|  | Ice source | Triage protocols (posters and cards) | Surge cache of high priority emergency medications |
|  | Primary and backup power supplies (wired or generator, solar augment) | Pill crusher | Surge cache of high priority emergency supplies |
|  | Primary and backup water sources (e.g., bladder, tank) and hosing if needed | Tablet cutter | Waterproof storage container |
|  | Non-sterile pad with impermeable barrier | Linens (e.g., sheets, pillowcases, towels, patient gowns) |  |
|  | Automated external defibrillator (AED) | Pillows |  |
|  | | | |
| **Register and Comunication (16)** | System for recording and executing orders | Medical records platform for documenting care (e.g., registration, substrate of QI, epidemiologic monitoring) | Medical records platform for documenting care (e.g., registration, substrate of QI, epidemiologic monitoring) |
|  | System to record care and provide transition instructions | Standardized clinical chart (e.g., pediatric, medical, trauma, obstetric) | Standardized clinical chart (e.g., pediatric, medical, trauma, obstetric) |
|  | Cards for communication number distribution | Registration form with patient contact information | Registration form with patient contact information |
|  | Important telephone number/radio frequency list | Medication instruction cards (non-word-based) | Medication instruction cards (non-word-based) |
|  | Condition specific protocols (posters or cards/book) | Printed book of disaster protocols (multiple copies for administrative and clinical areas) | White board |
|  | Integrated information management system (allows integration of information from multiple sources about single patient; medical record number) | Pre-printed disaster intake forms | In-unit communication system |
|  | Computer(s) | Shortwave radio or dedicated telephone line |  |
