# Supplementary material for: Assessment of critical resource gaps in pediatric injury care in Mozambique’s four largest Hospitals
Source: PLoS One. 2023 Jun 1;18(6):e0286288. doi: 10.1371/journal.pone.0286288 (PMC10234533; doi:10.1371/journal.pone.0286288)
Supplement: S2 Table — Note. From the medication checklist, was removed item nine about obstetric emergency units. (DOCX) [file pone.0286288.s002.docx]

**S2 Table:** Medications categories according to functions for essential trauma care, with reference to the WHO checklist

| **Categories for essential trauma care** | **WHO Checklist medication** | |
| --- | --- | --- |
| **Infections (16)** | Topical antifungals | Time sensitive prophylaxis after infectious exposure (including HIV) |
|  | Oral antifungal | Oral/IM antibiotics for lung, skin, GI, or GU source (including syndromic STI treatment, PPROM) |
|  | IV antifungal | IV antibiotics (for lung, skin, GI, GU, or CNS source; PPROM) |
|  | Oral antimalarials | Tetanus vaccine |
|  | IV antimalarials | Anti-tetanus immunoglobulin |
|  | Oral anthelmintics | Rabies vaccine |
|  | Oral antiviral (acyclovir or equivalent) | Anti-rabies immunoglobulin |
|  | IV antiviral (acyclovir or equivalent) | Time sensitive prophylaxis after infectious exposure (including HIV) |
|  | Ophthalmic topical antimicrobial | Oral/IM antibiotics for lung, skin, GI, or GU source (including syndromic STI treatment, PPROM) |
|  | | |
| **Poisoning (11)** | Antidote for lead exposure | Pyridoxine |
|  | Antidotes for iron overdose | Thiamine |
|  | Antivenin | Diphenhydramine |
|  | Antidote for cyanide toxicity | N-Acetylcysteine |
|  | Activated charcoal | Pyridoxine |
|  | Atropine | Thiamine |
|  | Neostigmine |  |
|  | | |
| **Cardiovascular disorders (10)** | Adenosine | IV antihypertensive agent |
|  | Advanced vasopressor support | IV Betablockers or CCB |
|  | Amiodarone | Mannitol |
|  | Nitroglycerin SL | Oral diuretics |
|  | IV Nitroglycerine | IV diuretics |
|  | | |
| **Anticoagulants and Thrombolytics**  **Hemostatic agents (4)** | Reversal agents for systemic anticoagulation | Thrombolytic (streptokinase for STEMI) |
|  | Systemic anticoagulation | Medical therapies for gastrointestinal bleeding |
|  | IM & IV antipsychotic | Antiepileptic other than benzodiazepine |
|  | Oral antipsychotic | IM & IV benzodiazepine |
|  | Oral & rectal benzodiazepine |  |
|  | | |
| **Corticosteroids (5)** | Agents for acute glaucoma (IV acetazolamide, ophthalmic topical steroid, ophthalmic topical beta-blocker) | IV steroids (for airway and CNS) |
|  | Ophthalmic topical steroids | Topical steroid |
|  | Oral steroids |  |
|  | | |
| **Fluids and electrolyte balance including blood (9)** | Oral rehydration solution | Oral (buccal) glucose |
|  | IV fluids for rehydration | IV glucose |
|  | Transfusion (whole blood, FFP, PRBC) | IV Calcium |
|  | IV potassium | Bicarbonate infusion |
|  | Oral potassium |  |
|  | | |
| **Skin diseases: topical applications (5)** | Skin and surface disinfectants | Topical anti-dermatome parasitic agent |
|  | Topical agents for ophthalmic exam (analgesics, mydriatics) | Topical agents for burn dressing |
|  | Other locally relevant treatments for bites and stings |  |
|  | | |
| **Analgesic, antipyretic anti-inflammatory (6)** | Oral aspirin | IV analgesia (opioid and non-opioid) |
|  | Oral & IM analgesia (opioid) | Local anesthesia for injection |
|  | Oral & IM analgesia (non-opioid) | Oral/rectal paracetamol |
|  | | |
| **Vitamins and minerals (5)** | Pyridoxine | Oral Vitamin K |
|  | Thiamine | Oral zinc |
|  | IV Vitamin K |  |
|  | | |
| **Anesthetics (3)** | IV paralytic (depolarizing and non-depolarizing agent) | Ketamine for IV/IM administration |
|  | Naloxone |  |
|  | | |
| **Others (6)** | Inhaled bronchodilator | Oral anti-emetic |
|  | Nebulized bronchodilator | IV anti-emetic |
|  | Oxygen | Sterile water for injection |
|  |  |  |

From the medication checklist, was removed item nine about obstetric emergency units.
