# Supplementary material for: Assessment of critical resource gaps in pediatric injury care in Mozambique’s four largest Hospitals
Source: PLoS One. 2023 Jun 1;18(6):e0286288. doi: 10.1371/journal.pone.0286288 (PMC10234533; doi:10.1371/journal.pone.0286288)
Supplement: S3 Table — (DOCX) [file pone.0286288.s003.docx]

**S3 Table. Opinions of the emergency unit clinical staff concerning obstacles and priorities to improve injury care as well as the preparedness of the unit, stratified by hospital and occupational group.**

| **Question** | **Maputo**  % | | **Beira**  % | | **Quelimane**  % | | **Nampula**  % | |
| --- | --- | --- | --- | --- | --- | --- | --- | --- |
| **(N=)** | Doctor  (105) | Nurse  (42) | Doctor  (56) | Nurse  (11) | Doctor  (26) | Nurse  (17) | Doctor  (27) | Nurse  (21) |
| **a) Obstacles** | | | | | | | | |
| Lack of equipment | 2.9 | 22.0 | - | 5.6 | - | 5.9 | - | 15.0 |
| The team has no skills | 1.0 | 7.3 | 3.6 | 5.6 | 3.9 | 17.7 | 3.7 |  |
| I don't have skills | 24.8 | 31.7 | 32.1 | 38.9 | 38.5 | 11.8 | 51.9 | 50.0 |
| Unit overcrowding | 14.3 | 19.5 | 5.4 | 27.8 | 3.9 | 11.8 | 3.7 | 25.0 |
| Lack of medications | 41.9 | 14.6 | 46.4 | 16.7 | 38.5 | 23.5 | 22.2 | 10.0 |
| **b) Priority** | | | | | | | | |
| Trauma courses | 3.8 | 7.5 | 10.7 | 15.8 | 15.4 | 17.7 | 3.7 | 15.0 |
| Trauma center | 29.5 | 50.0 | 35.7 | 47.4 | 57.7 | 47.1 | 40.7 | 60.0 |
| Increase drug supply | 12.4 | 10.0 | 10.7 | 10.5 | 7.7 | 17.7 | 3.7 | 10.0 |
| Service protocols | 21.0 | 12.5 | 33.9 | 26.3 | 11.5 | 11.8 | 37.0 | 10.0 |
| *Other* | 33.3 | 20.0 | 8.9 |  | 7.7 | 5.9 | 14.8 | 5.0 |
| **c) Emergency preparedness** | | | | | | | | |
| Unprepared | 36.9 | 23.1 | 33.3 | 47.4 | 30.8 | 30.8 | 59.3 | 50.0 |
| Prepared | 12.6 | 25.6 | 14.8 | 10.5 | 15.4 | 15.4 | 11.1 | 15.0 |
| Unorganized | 8.7 | 20.5 | 25.9 | 31.6 | 7.7 | 7.7 | 14.8 | 15.0 |
| Organized | 35.0 | 25.6 | 18.5 | 5.3 | 42.3 | 42.3 | 14.8 | 10.0 |
| Others | 6.8 | 5.1 | 7.4 | 5.3 | 3.9 | 3.9 | - | 10.0 |
